# Deferred autologous stem cell transplantation in systemic AL amyloidosis

**DOI:** 10.1038/s41408-018-0137-9

**Published:** 2018-11-05

**Authors:** Richa Manwani, Ute Hegenbart, Shameem Mahmood, Sajitha Sachchithanantham, Charalampia Kyriakou, Kwee Yong, Rakesh Popat, Neil Rabin, Carol Whelan, Tobias Dittrich, Christoph Kimmich, Philip Hawkins, Stefan Schönland, Ashutosh Wechalekar

**Affiliations:** 1 National Amyloidosis Centre, University College London, London, UK; 2 Medical Department V, Amyloidosis Centre, University Hospital Heidelberg, Heidelberg, Germany; 3 Department of Haematology, University College London Hospitals, London, UK

## Abstract

High-dose melphalan with autologous stem cell transplantation (ASCT) can induce durable haematological and organ responses in systemic AL amyloidosis (AL). Stringent selection criteria have improved safety of ASCT in AL but most patients are transplant-ineligible. We report our experience of deferred ASCT in AL patients who were transplant-ineligible at presentation but had improvements in organ function after induction chemotherapy, enabling them to undergo ASCT. Twenty-two AL patients underwent deferred ASCT from 2011 to 2017. All had serial organ function and clonal response assessment. Organ involvement and responses were defined by amyloidosis consensus criteria. All patients were transplant-ineligible at presentation, predominantly due to advanced cardiac involvement. All received bortezomib-based therapy, with 100% haematologic response (86% complete response (CR)/very good partial response (VGPR)), enabling reversal of ASCT exclusion criteria. Patients underwent deferred ASCT for haematologic progression (45%) or consolidation (55%). There was no transplant-related mortality. Haematologic responses post-ASCT: CR 50%, VGPR 27%, PR 18%, non-response 5%. In all, 85.7% achieved cardiac responses. Median overall survival (OS) was not reached. Median progression-free survival (PFS) was 54 months. This selected cohort achieved excellent haematologic responses, organ responses, PFS and OS with deferred ASCT. If larger studies confirm these findings, this may widen the applicability of ASCT in AL.

## Introduction

Systemic amyloid light chain amyloidosis (AL) is characterised by deposition of misfolded immunoglobulin light chains within organs, leading to severe visceral dysfunction. Cardiac involvement is a critical determinant of survival. High-dose melphalan with autologous stem cell transplantation (ASCT) in AL can result in high haematologic response rates, with over a third of patients achieving complete haematologic response^1,2^, translating into promising organ responses^3^. Response to ASCT appears to be durable with a median overall survival (OS) of 7.6 years reported by the Boston group^1^. Bone marrow plasma cell infiltration >10% prior to ASCT is a poor prognostic factor^4^. Transplant-related mortality (TRM) has historically been high (early reports of up to 40%) in unselected patients^5^. The recognition that advanced cardiac involvement is associated with higher TRM has led to refined patient selection strategies with a reduction in TRM to ~5%^2^ and most recently 2.4% reported by the Mayo group^6^. However, stringent selection criteria, which vary in each country, render the majority of patients with cardiac amyloidosis transplant-ineligible.

Modern chemotherapy agents can induce haematologic and organ responses in AL, including those with cardiac involvement, but durability of response remains uncertain^7–9^. No study has demonstrated the prolonged progression-free survival (PFS) patients treated with non-transplant regimes akin to that achieved with ASCT. It is now apparent that a proportion of patients with significant cardiac involvement will substantially improve after achieving a good response to chemotherapy. While studies have examined the role of bortezomib-based induction chemotherapy immediately prior to ASCT, no studies to date have specifically focussed on the role of deferred ASCT in transplant-ineligible patients^10–13^.

We therefore report a retrospective cohort of AL patients (from two large European amyloidosis centres) with advanced cardiac involvement who were considered transplant-ineligible at presentation but achieved haematological and organ responses with chemotherapy, allowing them to undergo ASCT later.

## Methods

All patients with AL ineligible for ASCT at presentation who had initial induction chemotherapy and then underwent deferred ASCT from September 2011 to July 2017 were included in this study. Reasons for transplant-ineligibility are listed in Table 2 and differed slightly between centres. Amyloidosis of AL type was confirmed on immunohistochemistry or by proteomic analysis of biopsy specimens. Exclusion of mutations in genes for hereditary amyloidosis was carried out as appropriate. All patients underwent serial protocolised assessment including biochemical tests for organ function, serum free light chain measurement, serum and urine protein electrophoresis and immunofixation, cardiac biomarker assessment, echocardiography and/or cardiac magnetic resonance imaging (unless contraindicated). Organ involvement and haematological response were defined as per international amyloidosis consensus criteria^14–16^. Progression was defined as haematologic progression, time to next treatment or death.

All patients underwent ASCT as per local protocols. Stem cells were mobilised with granulocyte colony-stimulating factor only in patients treated at the UK National Amyloidosis Centre and with cyclophosphamide priming in the patients at Heidelberg. Transplant conditioning was undertaken with intravenous melphalan, followed by infusion of autologous haematopoietic stem cells. Dose reduction of melphalan was used in patients with severe renal impairment. Standard institutional protocols were followed for post-transplantation supportive care and antimicrobial prophylaxis.

Primary outcome variables were TRM (defined as death within 100 days post-ASCT), haematologic and organ response, PFS and OS^15^. Statistical analysis was performed using SPSS version 24. Approval for analysis and publication was obtained from the institutional review boards, and written consent was obtained from all patients in accordance with the Declaration of Helsinki. Survival outcomes were analysed using the Kaplan–Meier method. All *p* values were two sided with a significance level of <0.05.

## Results

Twenty-two patients were included in this study. Nine were from Heidelberg University Amyloidosis Centre and 13 from the UK National Amyloidosis Centre. Baseline characteristics are presented in Table 1. The median age at presentation was 54 years (range 39–69 years). The number of patients with NYHA class 1, 2, 3 and 4 symptoms at presentation was as follows: 1 (4.5%), 17 (77.3%), 3 (13.7%), and 0, respectively. The number of patients with ECOG score 0, 1, 2, 3 and 4 was: 1 (4.5%), 12 (54.6%), 8 (36.4%), 1 (4.5%), and 0, respectively. The number of patients with Mayo cardiac stage I, II and III patients were as follows: 0, 3 (13.6%), and 19 (86.4%), respectively. Of the stage III patients, 2 patients had an N-terminal pro-brain natriuretic peptide (NT-proBNP) > 8500 ng/L (stage IIIb as per the European variation of the original Mayo staging system^17^). At diagnosis, median NT-proBNP and median troponin T were 2924 ng/L (range 624–28737 ng/L) and 62.5 ng/L (range 9–1885 ng/L), respectively. Baseline median left ventricular (LV) wall thickness was 15 mm (range 8–19 mm) and median LV ejection fraction was 55% (range 38–67%). The number of patients with renal, liver, soft tissue, peripheral nerve, autonomic nerve and gastrointestinal involvement was as follows: 8 (36.3%), 4 (18.2%), 9 (40.9%), 2 (9.1%), 2 (9.1%), and 5 (22.7%), respectively. The median baseline involved free light chains were 691.5 mg/L (range 135–9594 mg/L), with a median difference in involved and uninvolved light chains of 562 mg/L (range 118–6830 mg/L).

**Table 1:** Baseline characteristics

	At presentation	At ASCT	*p* Value
Median age (years)	54 (range 39–69)	56 (range 40–70)	<**0.001**
Male:female	15 (68%):7 (32%)		
NYHA class			
1	1 (4.5%)	13 (59.1%)	**0.0013**
2	17 (77.3%)	9 (40.9%)	
3	3 (13.7%)	0	
4	0	0	
Not recorded	1 (4.5%)	0	
ECOG			
0	1 (4.5%)	16 (72.7%)	<**0.0001**
1	12 (54.6%)	6 (27.3%)	
2	8 (36.4%)	0	
3	1 (4.5%)	0	
4	0	0	
Cardiac involvement	22 (100%)		
Median NT-proBNP (ng/L)	2924 (range 624–28,737)	415 (range 118–2853)	<**0.0001**
Median cardiac troponin T (ng/L)	62.5 (range 9–1885)	13 (range 5–76)	**0.0142**
Mayo stage			
I	0		
II	3 (13.6%)		
III	19 (86.4%)		
(Stage III patients with NT-proBNP > 8500 ng/L)	(2 (9.1%))		
Median systolic blood pressure (mm Hg)	114 (range 80–137)	114 (range 100–142)	0.2107
Median mean left ventricular wall thickness (mm)	15.0 (range 8–19)	14.0 (range 7.5–17.5)	0.0861
Median LV ejection fraction (%)	55 (range 38–67)	57 (range 39–65)	0.285
Renal involvement	8 (36.3%)		
Median creatinine (μmol/L)	77 (range 46–537)	80.5 (range 53–162)	0.697
Median GFR (mL/min)	97 (range 10–219)	88 (range 41–124)	0.313
Median proteinuria (g/24 h)	0.35 (range 0.1–4.7)	0.1 (range 0.1–4.1)	**0.0356**
Liver involvement	4 (18.2%)		
Median ALP (units/L)	75.5 (range 42–340)	70.5 (range 40–177)	0.2987
Soft tissue involvement	9 (40.9%)		
Peripheral nerve involvement	2 (9.1%)		
Autonomic nerve involvement	2 (9.1%)		
GI involvement	5 (22.7%)		
Median number of involved organs	2 (1–5)		
Kappa:lambda	9 (36%):13 (64%)		
Median involved FLC (mg/L)	691.5 (range 135–9594)	36.5 (range 0.2–950)	<**0.0001**
Median dFLC (mg/L)	562 (range 118–6830)	25.1 (range 1.6–911)	**0.0002**
Median serum monoclonal protein (g/L)	1 (range 0–16)	0 (range 0–6)	**0.0015**
Detectable serum paraprotein	8 (36%)	2 (9%)	**0.0011**
Serum paraprotein: light chain only/IgG/IgA/IgM/IgD	3 (17%)/5 (23%)/1(5%)/0/0		

*p* values less than 0.05 are found in bold text

All patients were regarded as transplant-ineligible at presentation, most commonly because of advanced, clinically significant cardiac involvement (reasons for transplant ineligibility are found in Table 2). All patients were treated with chemotherapy upfront. All patients had been treated with bortezomib-based therapy prior to ASCT. Treatment included bortezomib–dexamethasone in 5 (23%), cyclophosphamide–bortezomib–dexamethasone 13 (59%), bortezomib–lenalidomide–dexamethasone 2 (9%) and cyclophosphamide–thalidomide–dexamethasone 2 (9%), respectively. Seven (32%) patients switched chemotherapy after a median of three initial cycles of treatment (range 1–4) due to suboptimal initial haematologic responses. Of these seven patients, three went on to be treated with a bortezomib–alkylator–dexamethasone regime, three went on to be treated with a bortezomib–immunomodulatory–dexamethasone combination and one went on to have immunomodulator therapy. The median number of total frontline cycles was 6 (range 4–9). Haematologic responses after chemotherapy on an intent-to-treat (ITT) basis were: complete haematologic response (CR) 11 (50%), very good partial response (VGPR) 8 (36%) and partial response (PR) 3 (14%). At 6 months, 14 patients (64%) achieved a cardiac response, 5/8 (63%) with renal involvement achieved a renal response and 3/4 (75%) with liver involvement achieved a liver response^14^.

**Table 2:** Reasons for transplant ineligibility at presentation

Heidelberg exclusion criteria for ASCT	At presentation (*n* = 9)	At ASCT (*n* = 9)
Severe cardiac failure	6	0
ECOG PS ≥ 2	3	0
Systolic BP ≤ 90 mm Hg	2	0
Gastrointestinal bleeding	1	0

**UK NAC exclusion criteria for ASCT**	**At presentation (n** **=** **13)**	**At ASCT (n** **=** **13)**
NT-proBNP > 1000 ng/L	13	1 (patient had an NT-proBNP response after induction chemotherapy)
Systolic BP ≤ 90 mm Hg	1	0
Serum albumin < 20 g/L	1	0
eGFR < 40 ml/min	1	0
Large load on SAP scintigraphy	2	0
ECOG PS > 2	1	0

**Fig. 1:**
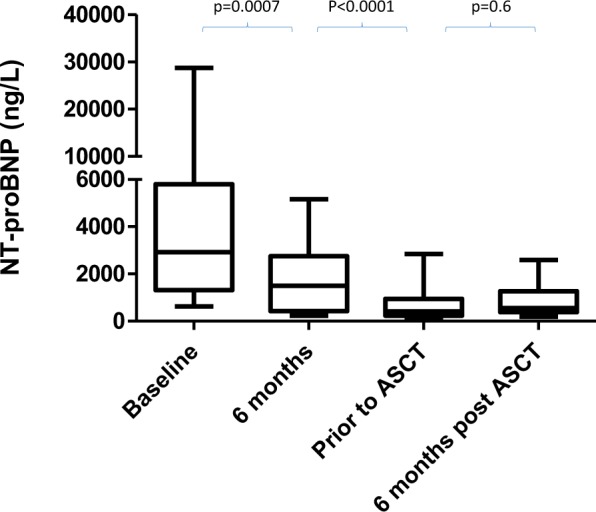
Serial median NT-proBNP measurements at baseline, 6 months post-diagnosis, prior to ASCT and 6 months post-ASCT (*n* = 22). Median NT-proBNP at baseline, 6 months post-diagnosis, prior to ASCT and 6 months post-ASCT were: 2924 ng/L (624–28737 ng/L), 1497 ng/L (range 415 ng/L (range 118–2853 ng/L), 415 ng/L (range 118–2853 ng/L), and 554 ng/L (range 186–2589 ng/L), respectively

The median time from presentation to ASCT was 14.5 months (range 6–45 months). The indication for ASCT was haematologic progression in 10 (45%) patients and as a consolidation procedure in the remainder. All patients had resolution of ASCT exclusion criteria, enabling them to undergo ASCT (Table 2). There were no serious adverse events or deaths during stem cell mobilisation. At the time of ASCT, median dFLC (difference between involved minus uninvolved serum free light chains) was 25.1 mg/L (range 1.6–911 mg/L). At the time of ASCT, all patients had NYHA class 1–2 symptoms compared to 81.8% at presentation (*p* = 0.0013) and Eastern Cooperative Oncology Group (ECOG) 0–1 prior to ASCT, compared to 59% of patients with ECOG 0–1 at presentation (*p* < 0.0001). The median NT-proBNP prior to ASCT was 415 ng/L (range 118–2853 ng/L), having been 2924 ng/L (624–28,737 ng/L) at presentation (*p* < 0.0001) and 1497 ng/L (237–5167 ng/L) 6 months after diagnosis (Fig. 1). The mean LV wall thickness was 15 mm (range 8–19 mm), compared to 14 mm (range 7.5–17.5 mm) at the time of ASCT (*p* = 0.086). The median LV ejection fraction at presentation was 55% (range 38–67%), compared to 57% (range 39–65%) at the time of ASCT (*p* = 0.285). Median cardiac troponin T was 13 ng/L (range 5–76 ng/L), having been 62.5 ng/L (range 9–1885 ng/L) at baseline (*p* = 0.0142). Median systolic blood pressure was 114 mmHg (range 80–137) at baseline and 114 mmHg (range 100–142) at the time of ASCT (*p* = 0.21). Median proteinuria had improved from 0.35 g/24 h (range 0.1–4.7) to 0.1 g/24 h (range 0.1–4.1) at ASCT (*p* = 0.036).

Median follow-up was 20.5 months (range 6–74 months). At 3 months post-ASCT, on an ITT basis, the number of patients in a CR, VGPR, PR and non-response was: 12 (54.5%), 7 (31.8%), 2(9.1%), and 1 (4.5%). The patient who had not achieved a haematological response to ASCT had undergone ASCT for haematological progression. The number of patients in a CR, VGPR, PR and non-response at 6 months post-ASCT on an ITT basis was: 11 (50%), 6 (27.3%), 4 (18.2%), and 1 (4.5%, died at 6 months) respectively. Cardiac responses were assessed in 21 patients (the remaining patient died at 6 months). Of these 21 patients, 18 (85.7%) had achieved a cardiac organ response compared to presentation. Eleven patients had an NT-proBNP <650 ng/L at the time of ASCT. Of the remaining ten patients, four patients had a cardiac response compared to NT-proBNP at the time of ASCT, four had no cardiac response compared to NT-proBNP at the time of ASCT and two had evidence of cardiac progression compared to NT-proBNP at the time of ASCT. Median NT-proBNP at 6 months post-ASCT was 554 ng/L (range 186–2589 ng/L) (Fig. 1). The median dFLC at the time of ASCT in the group who underwent ASCT for consolidation was 13 mg/L (range 1.6–36 mg/L). In the group who underwent ASCT for relapse, the median dFLC was 61 mg/L (range 24.3–911 mg/L), (*p* = 0.0002).

**Fig. 2:**
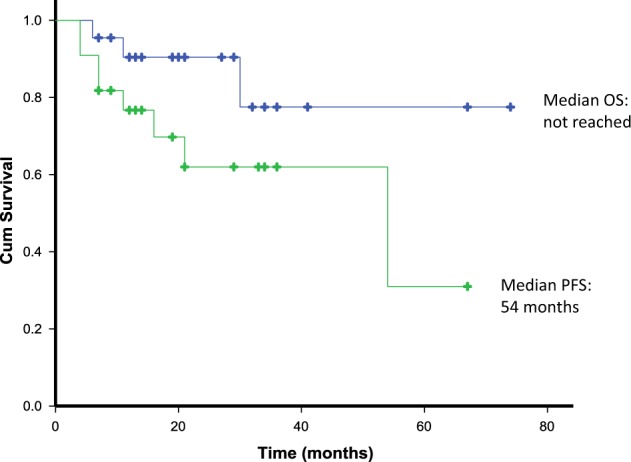
Overall survival (OS) and progression-free survival (PFS) from time of ASCT. Median OS from ASCT was not reached in the cohort. Median PFS of the cohort from time of ASCT was 54 months

**Fig. 3:**
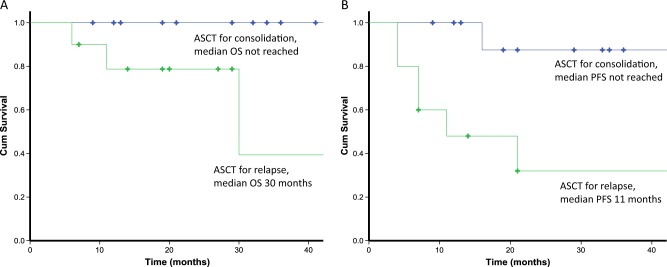
Overall survival (OS) and progression-free survival (PFS) in patients undergoing deferred ASCT for relapse or consolidation. **a** Median OS in patients who underwent deferred ASCT for consolidation was not reached; patients who underwent deferred ASCT for relapse had a median OS of 30 months. **b** Median PFS in patients who underwent for ASCT for consolidation was not reached; patients who underwent deferred ASCT for relapse had a median PFS of 11 months

There was no TRM in the cohort. The median OS of the cohort from time of ASCT has not yet been reached (Fig. 2). Three patients (13.6%) died (at 6, 11 and 30 months after ASCT). The cause of death in these patients was: congestive cardiac failure in 2 patients and chemotherapy-related sepsis (in a patient who developed haematologic progression 4 months after ASCT). Eight patients (36%) progressed after ASCT (defined as haematological progression, progression to next treatment or death). The median PFS in the cohort was 54 months (Fig. 2). The median OS and median PFS were not reached in the group who underwent ASCT for consolidation (Fig. 3). The median OS and median PFS were 30 months and 11 months, respectively, in the group who underwent ASCT for haematological progression.

## Discussion

ASCT can induce good haematologic and organ responses in systemic AL amyloidosis, as well as prolonged OS^1,6^. Historically, ASCT in AL has been fraught with a substantial risk of TRM^5^ often due to cardiac involvement, which is a key factor in patient selection^2^. However, given that most patients with AL have some degree of cardiac involvement, upfront ASCT is not a realistic treatment option for the majority of patients. We show in this cohort that patients with advanced cardiac involvement at presentation who were considered transplant-ineligible can be treated with chemotherapy, achieving haematological responses that enable improvements in organ function, allowing them to be safely transplanted at a later date.

ASCT remains an important treatment modality in AL since none of the standard chemotherapy regimens using novel agents have shown the prolonged PFS reported with ASCT. In patients with multiple myeloma, it is clear that ASCT still has a role in patients treated with novel agent-based combination regimes and increases the rate of minimal residual disease negativity, associated with longer PFS^18^. Since rigorous patient selection is key to achieving reduced transplant-related morbidity and mortality in AL, such selection strategies have become routine in assessment for ASCT. There remains no international consensus on specific exclusion criteria, but all centres concur that “significant” cardiac involvement is a contraindication to transplantation. In our centres, only 10–15% of all patients assessed are considered suitable for transplantation. Importantly, in this disease, mortality is concentrated in the early months after diagnosis when there is ongoing organ damage due to amyloidogenic light chain toxicity, along with toxicity from treatment regimens and ASCT. Mortality in this disease falls rapidly after the first 6–12 months, hence a deferred transplantation approach is attractive.

Previous studies have examined the role of ASCT later in the disease course. In the seminal study of 17 patients treated with bortezomib, cyclophosphamide and dexamethasone, Mikhael et al. demonstrated that this treatment regimen rendered three transplant-ineligible patients to become eligible for ASCT^13^. Cornell et al. went on to explore the role of bortezomib-based induction in 28 transplant-ineligible patients with AL^11^. A third of patients in the latter study achieved haematologic and organ responses with bortezomib-based therapy, subsequently enabling them to undergo ASCT. However, only four patients had advanced cardiac involvement that improved enough to enable transplant eligibility. A single-centre retrospective cohort study by Hong et al. demonstrated favourable outcomes in 20 patients treated with bortezomib-based therapy prior to ASCT, but only 10% had advanced cardiac involvement^12^. Similarly, Scott et al. studied outcomes in 31 patients with AL who underwent ASCT, of which 12 received bortezomib-based induction therapy^10^. The latter authors demonstrated superior overall haematologic and organ response rates in the bortezomib pre-treated group. Six patients were initially deemed transplant-ineligible but became eligible for ASCT after bortezomib-based therapy but only one patient had advanced cardiac disease at presentation. In a recent report of the Mayo group’s 20-year experience of ASCT in AL, 38% of patients underwent bortezomib-based treatment before ASCT in 2010–2016, compared to only 3% from 2003 to 2009. However, only a minority underwent ASCT >12 months after diagnosis and survival outcomes in Mayo stage (2012) III/IV cardiac involvement were poorer. It is unclear what proportion of patients who had deferred transplants had advanced-stage disease or achieved organ responses with induction chemotherapy prior to deferred ASCT^6^.

Unlike previous retrospective cohorts, patients in this cohort had Mayo stage II/stage III cardiac involvement at presentation (the majority with stage III involvement). After induction chemotherapy, 86% achieved a CR/VGPR and 64% achieved a cardiac response by international amyloidosis consensus criteria^15^. Median NT-proBNP fell from 2924 (624–28,737 ng/L) at presentation to 415 ng/L (range 118–2853 ng/L) prior to ASCT. ECOG score and NYHA class improved in the group, with all patients having ECOG score 0–2 and NYHA class 1–2 symptoms at ASCT.

Haematological responses 6 months post-ASCT were excellent, with 77.3% of patients achieving CR/VGPR on an evaluable basis. These translated into impressive cardiac organ responses—with 85.7% in a cardiac response 6 months post-ASCT. Despite the majority of patients having advanced cardiac involvement at presentation, there was no TRM in the group.

PFS and OS were significantly poorer in the group who underwent ASCT for haematologic progression compared to those who underwent ASCT as consolidation (Fig. 3). These findings are in contrast to an earlier retrospective study by one of our groups, which suggested that OS and PFS were not affected by timing of transplantation (i.e. ASCT at relapse or as consolidation)^19^. The latter study included patients from 2003 to 2012 and none of the patients received a bortezomib-based combination first line (having largely been treated with cyclophosphamide–thalidomide–dexamethasone or vincristine–doxorubicin–dexamethasone, compared to 91% patients in this study who received a bortezomib-based combination first line). It is possible that those patients who relapse having been previously treated with bortezomib-based therapy have poor risk disease, accounting for worse outcomes after ASCT compared to those who are treated for consolidation. Median dFLC at the time of ASCT were significantly higher in the relapse group compared to the consolidation group, and this may also account for inferior outcomes in the relapse group. Larger studies are essential to address the question of optimal timing of ASCT.

There is another important question of the role of ASCT in patients who are refractory to first-line bortezomib-based therapy. Given the relatively conservative approach to ASCT at our centres, we routinely do not undertake ASCT in patients who are refractory to therapy. Such patients proceed to second-line non-ASCT chemotherapy options to aim for deeper clonal responses. Seven patients in this cohort did not respond to initial chemotherapy and switched to second-line treatment (predominantly with bortezomib-based therapy) and achieved haematological responses prior to ASCT.

This retrospective small study needs to be interpreted within its limitations. It features a carefully selected patient cohort and does not capture ‘all-comers’ to our centres. Other experienced transplant centres may have deemed a proportion of patients included in this study to be suitable for upfront transplants. However, centres with lesser experience still need to be extremely cautious in cardiac AL patients due to the risk of TRM.

In conclusion, our data highlight that deferred ASCT can be undertaken safely in a consolidation or haematologic progression setting in selected patients with advanced cardiac AL who are initially transplant-ineligible but go on to achieve organ responses. The approach of novel agent-based chemotherapy followed by deferred ASCT may allow a greater proportion of patients with systemic AL amyloidosis to undergo ASCT. Larger studies with longer follow-up are required to confirm our findings, assess durability of response and clarify optimal timing of ASCT.
